# A conversation with Elizabeth Jaffee

**DOI:** 10.1172/JCI172246

**Published:** 2023-07-03

**Authors:** Ushma S. Neill

Elizabeth Jaffee, the Johns Hopkins–based oncologist and immunologist, led the clinical development of a first-gen cancer vaccine for pancreatic cancer and continues to use innovative approaches to identify the complex signaling pathways in tumor cells, the microenvironment, and the immune system toward the generation of cancer immunotherapies. All the while, Jaffee (Figure 1) has been a leader at the local, national, and international levels and currently is the chair of President Biden’s Cancer Panel. For the full interview, see www.jci.org/videos/cgms.

*JCI*: Can you tell us a little bit about your family?

Jaffee: I have humble beginnings; both my parents were children born during the Depression. My father was from an immigrant family from Russia, and my mom’s family had been here for a generation. My mother and father met when she was 19 and he was 22. My father had worked his way through college and my mom did one year of college; I’m the first woman in my family to have gone to college. My dad trained as an accountant and then was drafted into the army. When he came back, they had me — I’m the oldest of five — and then he started his own business in real estate. His uncle had given him a very small investment to buy a house in a poor neighborhood in the Bronx. My dad rehabbed it and rented it out.

We had moved to a two-family home in Canarsie, Brooklyn. My father unfortunately got shot while collecting rent in his building in the Bronx. It was traumatizing. In the mid-60s, I guess surgical techniques were not very good, so he was laid up for about a year. My mom had to go back to work. Our family now had four children, so it was very stressful. Eventually he got better, and his business took off again. We moved to Long Island, where we could attend better public schools. High school came around, and I put a lot of my efforts into schoolwork and activities. The one light in my life was that the second half of senior year, I met my soon-to-be husband; we were high school sweethearts who went to the prom together. We both ended up in Boston for college.

*JCI*: What were the roots of your scientific and medical interests?

Jaffee: When I was about eight, a beloved great-uncle developed cancer. His name was Happy, and I just couldn’t imagine how somebody with the name of Happy could die of cancer; it made me pay attention to science and medicine. Beyond that, I had a fourth-grade math teacher who noticed I was very good in math. She talked a lot about the race to the moon, and I also read Marie Curie’s biography, and to me, that was a bright light. Later, I had a chemistry teacher in eleventh grade, and the passion that came out from her was infectious. She encouraged me, and I thought chemistry was a lot of fun. I love chemistry, but I really wanted to go into the biological sciences. I chose Brandeis because it was one of the top biochemistry undergrad programs in the country.

I was able to work in the lab of a young assistant professor who was doing immunology. At that time, a major advance had been made: hybridoma technology. We came up with a project where I would develop hybridomas from immunized murine B cells and then fuse them and screen to identify a heavy chain switched antibody; we found a number. It was a great learning experience.

David Baltimore came and gave a talk to one of my classes when I was a sophomore as well. His talk about virology was so exciting. At that point, I realized how powerful the immune system can be against viruses. I’d always thought the two greatest biomedical achievements in the 20th century were penicillin/antibiotics and vaccines that prevent kids from dying in childhood. I started putting two and two together and thinking that if the immune system can see a virally infected cell, why can’t the immune system see a cancer that looks different from a normal cell? That got me started on my pathway toward immunotherapy and cancer.

*JCI*: Was medical school the natural next step?

Jaffee: I’d done some volunteer work with kids with cancer, and I liked the clinical aspects of that work. My husband and I decided to get married and went to New York because there were the most medical schools there where we both could potentially be accepted. I went to New York Medical College; he went to Columbia. I was able to train at two wonderful hospitals: one in Greenwich Village that took care of patients from all different areas of NYC, including prisons; the other was Metropolitan Hospital in Harlem where by and large the patients were poor and did not have the best access to care. I was in medical school from 1981 to 1985 in New York City, so we were there for the burgeoning of the HIV/AIDS epidemic. The hospitals were overwhelmed, and nobody understood the disease initially. I watched it unfold, and within a few years, we saw how quickly drugs were developed against this virus.

My husband and I wanted to be internists, and so we decided we had to leave New York to get experience elsewhere that was not dominated by AIDS. There was no couples match at that time, but the chair of medicine at University of Pittsburgh (Gerald Levy) offered to give us the same two weeks’ vacation every year, which many programs could not commit to at that time. Also, Gerry offered me a fourth year that was similar to the first year of a PhD where I was able to rotate through different labs and take didactic courses to learn different skills. I also conducted a yearlong research project. That’s when I met John Kirkwood, the melanoma immunobiologist. I ended up doing a longitudinal project with his team. After this year of research and realizing that we had no good cancer treatments (this was the mid to late 1980s), I realized I wanted to do a research fellowship. That led me to Johns Hopkins.

My husband was looking to do a master’s in epidemiology; we matriculated in 1989 and have never left. Part of that is because when I was a second-year fellow, I was looking for a plasmid and after looking at the literature I realized Dan Nathans here at Hopkins had it. I didn’t want to bother Dan Nathans — the 1978 Nobel Prize winner. But I called his assistant and 10 minutes later I got a call from him directly: “Dr. Jaffee, I’d like to hear about your project, tell me about it. And then I could tell you what you may need because I have a number of these plasmids.” That has been my experience here my whole career. It didn’t matter that I was just a fellow, I was treated as if I was a valued colleague.

This culture is also how I got into pancreatic cancer. I was working on genetically engineered tumor vaccines; Drew Pardoll and a few of us at Hopkins were working with researchers at the Whitehead at MIT to develop the ability to transduce tumor cells to express cytokines. And then I developed a human vaccine together with a kidney cancer surgeon, Fray Marshall. Both renal cancer and melanoma were cancers that were thought to be immunogenic, so it made sense to start there. I needed a lot of tumor cells to develop the human vaccine, and Fray would give me whatever pieces of tumors he was resecting that I needed. I realized science was fun and impactful. We were having a good time and very quickly developed the vaccine; we tested it in patients and had some evidence of activity. Based on that success, Bert Vogelstein recruited me to develop a vaccine for pancreatic cancer as part of his GI SPORE proposal. There was some clinical research on pancreatic cancer, but there was nothing innovative really coming to the clinics on a regular basis, and so this was my opportunity. Additionally, my father’s brother had died of pancreatic cancer when I was a resident. So pancreatic cancer became my calling.

*JCI*: It sounds like it was this perfectly linear upward trajectory of success, but I imagine it was not that easy.

Jaffee: Early on, when I had developed the genetically engineered cancer vaccine, it was only the gene-therapy community that embraced us. At the bigger meetings like AACR or ASCO, yes, we were invited to speak, but we were usually the last session of the last day. Nobody showed up except a few other immunologists. But there was a rapid transformation as the immune checkpoint drugs were making their way to the clinics. We all knew about these agents and their efficacy in mice, but the clinical world hadn’t really embraced immunology. I had repeatedly heard, “Immunology will never be a therapy for cancer patients.” Then Jim Allison started presenting patients who were responding to anti–CTLA-4, and all of a sudden, we went from night to day. Immunotherapy was finally viewed as a potential clinical modality for cancer treatment.

*JCI*: Layered on top of the science, you’ve taken on some rather meaty leadership roles.

Jaffee: I made the decision early on that I wanted to give back to the NCI. I was invited to be on a program committee and a parent committee and then joined the board of scientific counselors, which investigates all the labs at the NCI. I learned how the NCI works.

I was also working my way up at AACR and elected to lead the Cancer Immunology Task Force — this was right before anti–CTLA-4 was approved. Harold Varmus, who is a wonderful person but did not appreciate immunology at the time, was leading the NCI. I decided to take five immunologists to his office and essentially said, “You realize that the ship is going to sail, and the NCI won’t be able to take any credit?”

I guess he put my name on a list; he could have hated me. But he clearly recommended me to be appointed to the National Cancer Advisory Board. And the next thing I knew, Obama’s administration called me. At that point, Varmus decided to leave NCI and Doug Lowy took over. At the next meeting, Doug gave us an overview of priorities. Anti–CTLA-4 was approved, PD-1 was on its way, but after 45 minutes, I remarked to Doug that he had not once mentioned cancer immunology. After that, I remained on the lists, even if I was outspoken, maybe because of it.

The opportunity for the Blue Ribbon Panel came along, and Vice President Biden was going to lead the moonshot. Myself, Tyler Jacks, and Dinah Singer led this committee, meeting often with Vice President Biden. At one point, I ended up organizing a meeting for Katie Couric and Vice President Biden, and we met with him in his office at the White House. This is when he was being asked to run for president, and he mentioned his promise to his son about curing cancer. It was so genuine and really hit me hard. I had lost my husband to ALS at that point, and it was as gutting as the cancers that I studied — maybe worse because there was no hope. I never understood the word hope before as well as I do now. My husband had no hope. Vice President Biden had no hope for his son, but he had hope for other people, and that was a connection we both made with each other.

Tyler, Dinah, and I presented the priorities we coalesced around, and I had to testify at Congress—Tyler and I testified, and that was quite an experience. Then President Obama appointed me as the NCAB chair; I think I’m the first woman chair of the National Cancer Advisory Board. I ended up staying on it for quite a while, including when the pandemic happened.

I did get asked to be considered for NCI director early on, to be considered for NIH director, and for NCI director again, and my answer has always been that I love working with all of them. I love the people at the NCI, but I also love my job. I love mentoring. I love research. We’re at a critical time in pancreatic cancer research. I want to be a part of the progress that must be made soon. I feel like my impact is different than what it would be if I took more of an administrative job.

*JCI*: If you had to do it over again, what other realm do you think could have kept you this passionate?

Jaffee: After having kids, I’d be a teacher. Not just teaching math, but developing new programs, really thinking about how we teach children as they mature based on what we know about child development, based on what we know about new technologies. I would love to be involved in developing programs that would help young people develop to the best of their abilities.

## Figures and Tables

**Figure 1:**
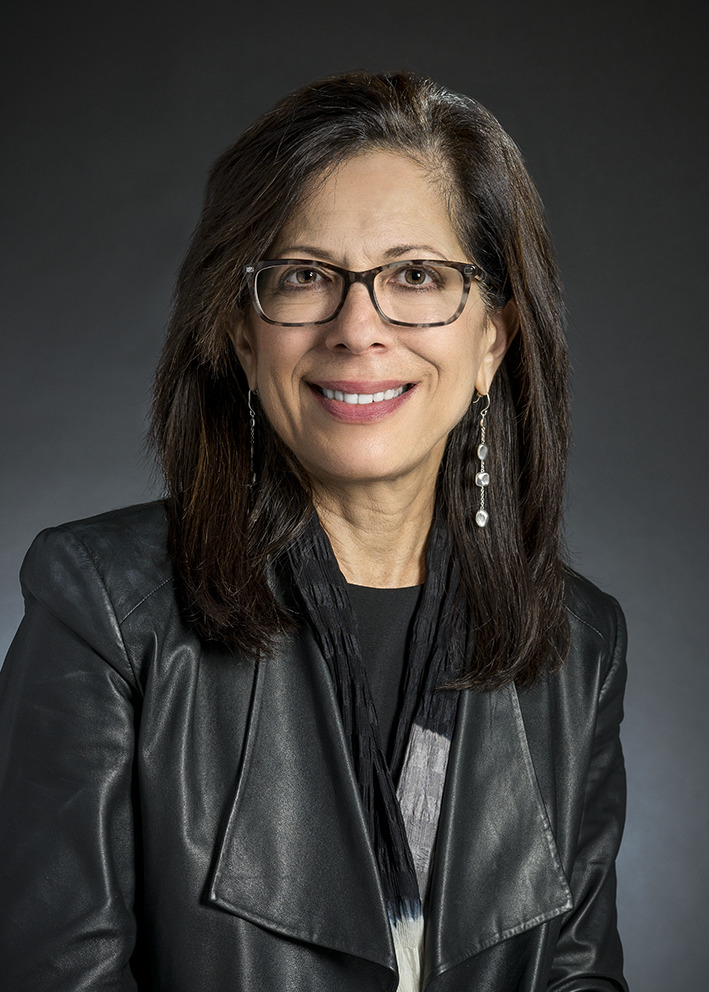
Elizabeth Jaffee.

## Version 1 07/03/2023

Electronic publication

